# Few-shot crop disease recognition using sequence- weighted ensemble model-agnostic meta-learning

**DOI:** 10.3389/fpls.2025.1615873

**Published:** 2025-08-06

**Authors:** Junlong Li, Quan Feng, Junqi Yang, Jianhua Zhang, Sen Yang

**Affiliations:** ^1^ School of Mechanical and Electrical Engineering, Gansu Agricultural University, Lanzhou, China; ^2^ Agricultural Information Institute, Chinese Academy of Agricultural Sciences, Beijing, China; ^3^ National Nanfan Research Institute, Chinese Academy of Agricultural Sciences, Sanya, China

**Keywords:** crop disease recognition, few-shot learning, meta-learning, ensemble learning, sequence-weighted ensemble

## Abstract

Diseases pose significant threats to crop production, leading to substantial yield reductions and jeopardizing global food security. Timely and accurate detection of crop diseases is essential for ensuring sustainable agricultural development and effective crop management. While deep learning-based computer vision techniques have emerged as powerful tools for crop disease recognition, these methods are heavily reliant on large datasets, which are often difficult to obtain in practical agricultural settings. This challenge highlights the need for models capable of learning from limited data, a scenario known as the few-shot learning problem. In this paper, we introduce a novel few-shot learning approach, the Sequence-Weighted Ensemble Model-Agnostic Meta-Learning (SWE-MAML), designed to train crop disease recognition models with minimal sample sizes. The SWE-MAML framework employs meta-learning to sequentially train a set of base learners, followed by a weighted sum of their predictions for classifying plant disease images. This method integrates ensemble learning with Model-Agnostic Meta-Learning (MAML), allowing the effective training of multiple classifiers within the MAML framework. Experimental results show that SWE-MAML demonstrates strong competitiveness compared to state-of-the-art algorithms on the PlantVillage dataset. Compared to the original MAML, SWE-MAML improves accuracy by 3.75%–8.59%. Furthermore, we observe that the number of base learners significantly influences model performance, with an optimal range of 5–7 learners. Additionally, pre-training with a larger number of disease classes enhances the model’s ability to recognize “unseen” classes. SWE-MAML was also applied to a real-world few-shot potato disease recognition task, achieving an accuracy of 75.71% using just 30 images per disease class in the support set. These findings validate that SWE-MAML is a highly effective solution for the few-shot recognition of crop diseases, offering a promising approach for practical deployment in agricultural settings where data scarcity is a major challenge. The integration of ensemble learning with meta-learning enables high-performance disease recognition with minimal data, marking a significant advancement in the field.

## 1 Introduction

Food security problems caused by crop diseases need to be solved urgently (Burdon et al., 2020; Ristaino et al., 2021). Globally, diseases cause losses of crop yield up to 40%, with the mean ranging from 30.3% (24.6-40.9%) in rice, 22.6% (19.5-41.4%) in maize, 21.5% (10.1-28.1%) in wheat, 21.4% (11-32.4%) in soybean, and 17.2% (8.1-21.0%) in potato (Savary et al., 2019). Consequently, accurate recognition and detection of crop diseases are necessary and pressing. Currently, crop diseases are mainly diagnosed manually by plant protection experts (Li et al., 2021). This traditional manual visual observation method is time-consuming and laborious, and subjective. For less experienced farmers, once the diagnosis is wrong, the best time for disease control will be missed. More importantly, misdiagnosis can lead to drug abuse, which fails to control disease and can cause pesticide residues, aggravate environmental pollution, and damage natural ecosystems. It follows that accurate, rapid, and intelligent crop disease recognition is becoming crucial.

Disease diagnosis can be automated using computer vision techniques (Ngugi et al., 2021). In the past decade, advancements in deep learning have brought about profound transformations in the field of plant disease research. Convolutional Neural Networks (CNNs), as the predominant network architecture in the field of disease analysis, have been extensively studied in various plant disease detection tasks and have demonstrated high recognition rates. In recent years, the Vision Transformer (ViT) architecture has also garnered widespread attention. Both CNN and ViT models have shown outstanding performance in the classification, detection, and segmentation of plant diseases (Ahmad et al., 2023; Mustofa et al., 2023; Shoaib et al., 2023; Pacal et al., 2024). However, traditional CNNs-based disease recognition relies heavily on massive amounts of training data and high computing power. If the amount of training data is too small, the classification models will not be able to learn the disease’s features effectively, leading to a significant weakening of the accuracy. Transfer learning is often employed to improve the disease recognition accuracy when there are insufficient samples. In this case, a pre-trained model is learned from the large dataset and then transferred to the classification task on the specific disease dataset (Simhadri and Kondaveeti, 2023; Khan et al., 2024). However, this method still relies on a medium-sized database in the fine-tuning phase. As a result, it does not accurately identify diseases when there are only a few to dozens of samples. In practice, obtaining and labeling disease samples from the field is extremely challenging (Xu, 2022). Agricultural scenarios are usually complex and diverse. Crop disease occurrence has a long time and space span in general. The same disease may exhibit different statuses at different growth stages and under different cultivation environments. As a result, it is difficult to collect all the features of a specific crop disease on a large scale in agricultural production. Furthermore, crop diseases spread widely in time and space, and the annotation of disease data needs to be done manually by experienced experts. Therefore, large-scale disease image collection and annotation is very costly.

Recent research in machine learning has demonstrated that few-shot learning (FSL) exhibits effectiveness for plant disease classification over small datasets (Yang et al., 2022; Porto et al., 2023), which may provide a promising scheme for solving the problems above. As the name implies, few-shot learning refers to feeding a learning model with a tiny amount of training data, contrary to the standard practice of using a large amount of data. Few-shot learning for image recognition can be mainly grouped into model initialization, metric learning, and data augmentation. Model initialization-based methods aim to learn a good set of initial values for fast adaptation to the novel classes (Finn et al., 2018). For metric learning-based methods, the classification is performed by calculating the similarity of image features in the few-shot learning tasks, such as cosine similarity (Vinyals et al., 2016), ridge regression (Bertinetto et al., 2019), graph neural networks (Kim et al., 2019), and multi-contrastive loss (Argüeso et al., 2020; Li and Yang, 2020). Regarding data augmentation-based methods, the key is how to train a generator that can generate synthetic samples (Hariharan and Girshick, 2017). Sometimes, the generator could be replaced by generative adversarial networks (ZHANG et al., 2018). The researchers have begun to apply few-shot learning to disease recognition. Egusquiza et al. (2022) suggested a deep metric learning-based method to extract latent space representations from crop diseases with just a few images using a Siamese network and triplet loss function. Yan et al. (2023) deep transfer learning framework adapting mixed subdomains was proposed, which effectively captures fine-grained target information and yields better results when dealing with poorly correlated subdomains. Xie et al. (2023) proposed the Salp Swarm Algorithm-based feature selection strategy effectively reduces feature dimensionality while enhancing classification success, with experimental results across multiple datasets validating its superior capability in selecting effective features for plant disease image classification. Javidan et al. (2024) introduced a Few-Shot Learning method based on Cosine Similarity, which was successfully applied to tomato disease image recognition. In essence, their algorithm is still a transfer learning.

Model-Agnostic Meta-Learning (MAML) is one of the most popular few-shot learning algorithms (Finn et al., 2017). Due to its flexibility, it can incorporate various model architectures and be applied to different problems. MAML is a meta-learning framework based on model initialization by training the model’s parameters so that a small number of gradient updates are going to lead to fast learning on a novel task. However, it comes with the need for costly hyperparameter tuning for training stability, and its performance has fallen behind many recent algorithms nowadays. Some researchers have presented modified versions to improve MAML, e.g., MAML++ (Antoniou et al., 2019), CAVIA (Zintgraf et al., 2019). In this paper, we propose a novel framework that introduces a strategy of MAML combined with ensemble learning, which performs well in few-shot crop disease classification. Ensemble learning is a machine learning paradigm where multiple learners are trained to solve the same problem (Dong et al., 2020). In contrast to ordinary machine learning approaches, which try to learn one hypothesis from training data, ensemble methods try to construct a set of hypotheses and combine them. It has been widely applied to many tasks, such as image recognition (Bu et al., 2024), active learning (Hessmann et al., 2025), and out-of-distribution robustness (Shen et al., 2024). Compared to a single model, an ensemble of several models may be more efficient and robust (Kondratyuk et al., 2020; Wenzel et al., 2020). How to combine MAML with ensemble learning is not explicit. Firstly, it is important to note that MAML is a strategy for training a learner (model) and not the learner itself. Secondly, MAML is a FSL method of model initialization, and its output is a meta leaner with the initialization parameters of the model for subsequent training. In the case of multiple learners (models) within ensemble learning framework, a direct idea is to use MAML to train initialization parameters for these learners independently in the first phase. These learners are then incorporated into the ensemble learning framework for retraining with respective initialization parameters for the specific task in the second phase. However, in the second phase of FSL, available samples are very sparse. The weights of each learner can only be trained on a few samples. This limited training prevents the weights from being fully trained and used in conjunction with fully trained learners. In the proposed method, we embed ensemble learning into the training process of MAML. Under the frame of MAML, we train the base learners in sequence along with the corresponding weights simultaneously. Since the models and the weights are trained on a large number of tasks in the first stage of MAML, they can be fully trained. This synchronous training enhances the fitness between the learner and its weights, resulting in improved recognition accuracy. In addition, the proposed method outputs only one meta learner, which saves the cost of model storage. In the training process, we only initialize the first base learner with the meta learner, and the initial parameters of other base learners can be obtained from the previous base learner. Due to this characteristic of training in sequence and weighting multiple learners, we call the proposed algorithm sequence-weighted ensemble of MAML (SWE-MAML). In our experiments, SWE-MAML shows good performance, which defeats the other comparison FSL algorithms thanks to the combination of ensemble learning and meta-learning.

The main contributions of this work are three-fold:

1. We propose a novel FSL method SWE-MAML to recognize few-shot crop diseases, which embed ensemble learning into frame of MAML to train multiple learners and their weights in sequence. Comparing with the existing related FSL, our method has achieved at the best accuracy.
2. We investigate the effect of the number of base learners and the number of source domain categories on the performance of few-shot classification by extensive comparative experiments over PlantVillage.
3. We explore the performance of SWE-MAML for few-shot disease recognition over the potato disease database of natural scenes. The algorithm still maintains high recognition accuracy when the target domain is very different from the source domain.

The rest of this paper is structured as follows. Section 2.1 presents the dataset used to develop and validate the algorithms in this study. Section 2.2 describes in detail the process and principles of implementing the method proposed in this study. Section 3 conducts extensive experiments and describes the details and results. The section includes the effect of the number of base-learners and the number of pre-trained classes on the model recognition accuracy, a comparison of the performance of FSL state-of-the-art models in crop disease recognition tasks in recent years, and an application of the proposed method to the potato disease recognition task under natural conditions. Finally, this work is discussed and concluded in Section 4 and 5.

## 2 Materials and methods

### 2.1 Materials

**Figure 1:**
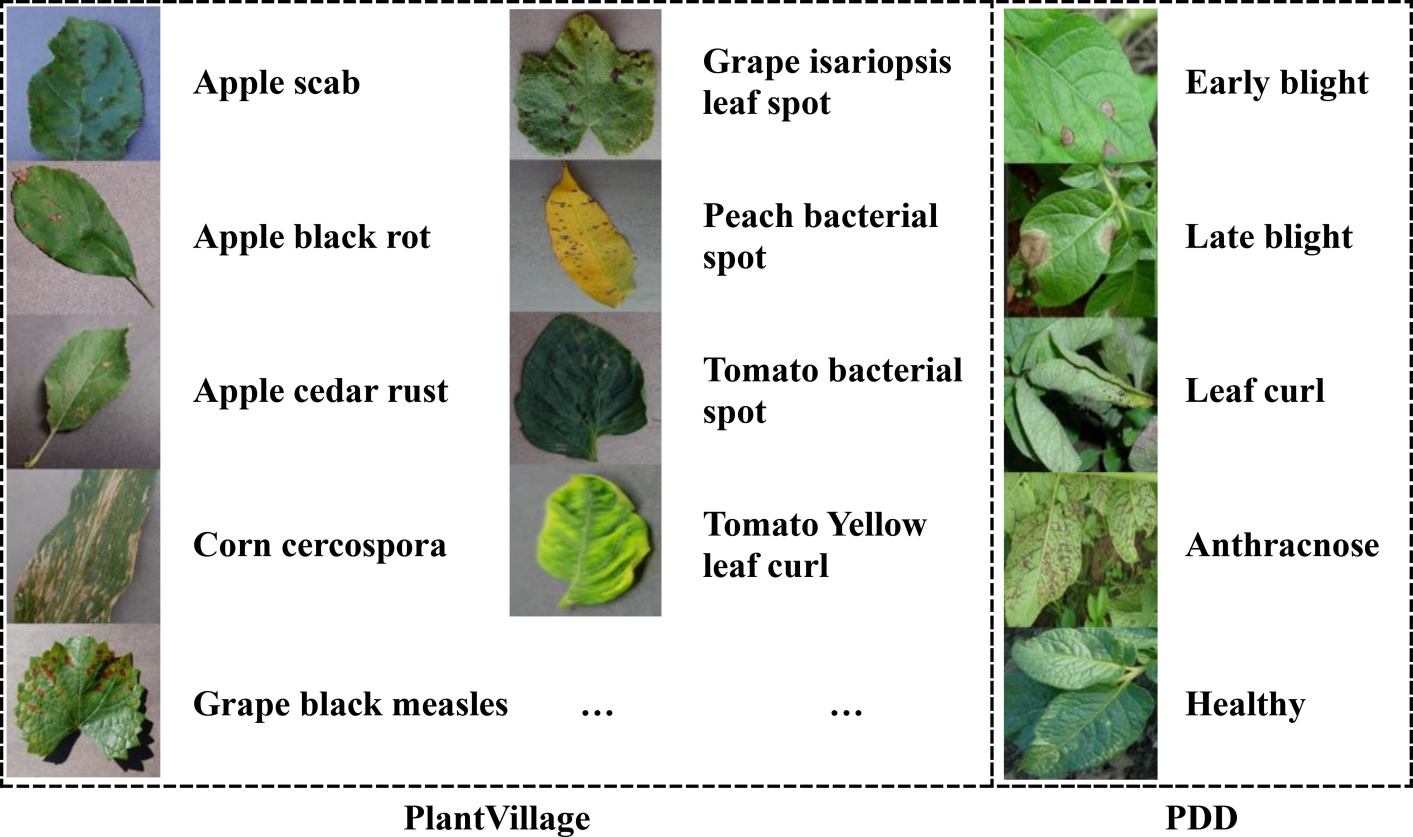
Some examples of the PlantVillage and PDD.

In this study, we employ two crop disease datasets to test the performance of our algorithm. The first is PlantVillage (Hughes and Salathe, 2016), an open-access repository of images on crop diseases. It collects 54,306 images of crop disease leaves, containing 14 kinds of crops and a total of 38 categories. All dataset images are taken under laboratory conditions with controlled lighting and simple backgrounds. **Table 1** displays the types of crops and their diseases. Some examples of the PlantVillage are given in **Figure 1**. All the image’s size is 256*256*3, corresponding to the RGB channels.

**Table 1:** Information of categories and images in PlantVillage.

Class	Name	Disease	Images	Class	Name	Disease	Images
C01	Apple	scab	630	C20	Pepper	healthy	1478
C02		black rot	622	C21	Potato	early blight	1000
C03		cedar rust	275	C22		healthy	152
C04		healthy	1645	C23		late blight	1000
C05	Blueberry	healthy	1502	C24	Raspberry	healthy	371
C06	Cherry	healthy	854	C25	Soybean	healthy	5090
C07		powdery mildew	1052	C26	Squash	powdery mildew	1835
C08	Corn	cercospora	513	C27	Strawberry	healthy	456
C09		rust	1192	C28		leaf scorch	1109
C10		healthy	1162	C29	Tomato	bacterial spot	2127
C11		northern leaf blight	985	C30		early blight	1000
C12	Grape	black rot	1180	C31		healthy	1591
C13		black measles	1383	C32		late blight	1909
C14		healthy	423	C33		leaf mold	952
C15		isariopsis leaf spot	1076	C34		septoria leaf spot	1771
C16	Orange	citrus greening	5507	C35		spider mites	1676
C17	Peach	bacterial spot	2297	C36		target spot	1404
C18		healthy	360	C37		mosaic virus	373
C19	Pepper	bacterial spot	997	C38		Yellow leaf curl	5357

The second dataset is the Potato Disease Dataset (PDD). Unlike the PlantVillage dataset, all PDD images are captured under natural lighting conditions with complex backgrounds. This dataset was constructed by querying and downloading publicly available photos from agricultural research websites and open-access image repositories between January and March 2024. To ensure diversity, samples were selected from at least five distinct geographic regions and four lighting conditions (full sun, partial shade, overcast, and backlit). The PDD comprises five diagnostic categories – Early Blight, Late Blight, Leaf Curl, Anthracnose, and Healthy Leaf – containing 40 images per class.

All downloaded images were processed through a two-stage quality control pipeline. First, images with resolution lower than 800×600 pixels or excessive blur (Laplacian variance threshold<100) were automatically filtered. Second, two certified plant pathologists independently annotated each retained image, with discrepancies resolved by a third expert, achieving a Cohen’s κ inter-annotator agreement of 0.87. Finally, all images were standardized through center-cropping (removing 10% border regions) and resizing to 256×256×3 resolution while preserving RGB channel integrity. Representative samples are shown in **Figure 1**.

In few-shot learning, the data is rare, so it is insufficient to constrain the problem. One possible solution is to gain experience from other similar problems, and most approaches characterize this strategy as a meta-learning problem (Wang et al., 2020). In the classical learning framework, the task is usually to learn how to classify from training data and evaluate the results using test data. Under the meta-learning framework, the task is learning how to learn to classify a given set of training tasks and evaluate using a set of test tasks. In other words, prior knowledge is learned from the source domain of classification problems to help solve the target domain of unrelated sets. To evaluate the transferability of SWE-MAML between the source domain and the target domain, we arrange two scenarios in our experiments: A. the source and the target come from PlantVillage, and B. the source comes from PlantVillage while the target comes from PDD. In Scenario A, PlantVillage is split into two parts without overlap, with one as the source domain and the other as the target domain. We evaluate several facts that influence the performance of SWE-MAML and compare the recognition accuracy of SWE-MAML and the other several algorithms. Although the target and the source domain have different disease categories, the images in them are taken under the same conditions. Thus, the feature distributions of the two domains are similar. In Scenario B, we further evaluate the transferability of our algorithm between the two domains with totally different feature distributions.

Few-shot learning is generally regarded as an N-way K-shot problem. N refers to the number of categories contained in the support set of each task, and K refers to the number of samples in each category. The support set is a small dataset used in the training phase, which generates prior knowledge for the testing phase. The query set represents the task on which the model actually needs to predict. Please remember that the query set’s categories never appear in the support set. In the meta-learning paradigm, when training the meta-learner with a set of tasks, it samples not only the data space but also the task space. Constantly adapting to each specific task enables the network to have an abstract learning ability. In Scenario A, we are randomly picking up N categories and K samples per category from the source or target domain for each task, and then the whole task has only N * K samples. K is generally not more than 20. In Scenario B, each task is created similarly to Scenario A.

**Figure 2:**
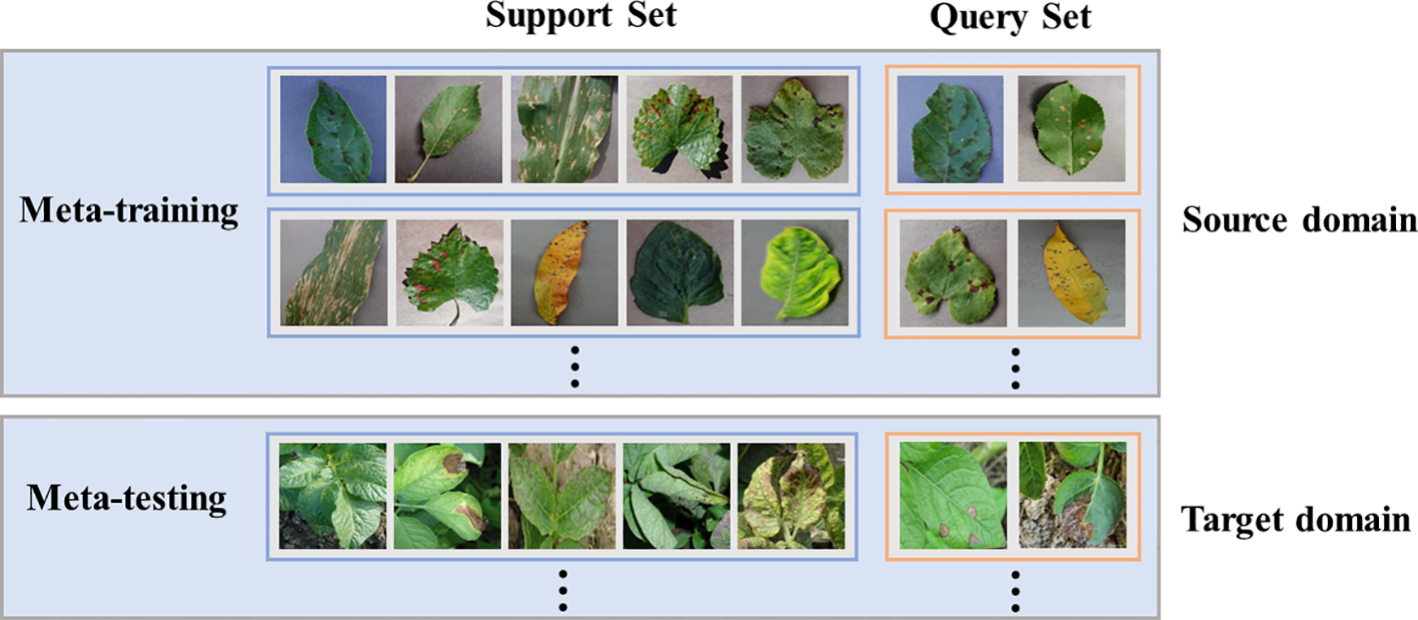
Schematic diagram of meta-learning.

Complete meta-learning usually includes two stages: meta-training and meta-testing. In most cases, a learner can be crystallized as a CNN. In the first stage, the meta-learner is trained, and prior knowledge is represented as the weights of the CNN. Thus, in the second stage, a CNN is created with the same structure and initialized by the meta-learner, and the same sampling mode of the N-way K-shot must be maintained. **Figure 2** gives the diagram of meta-learning.

### 2.2 Methods

The few-shot learning method refers to the model learning from a small amount of data. It can be implemented in a variety of ways, including matching networks (Vinyals et al., 2016), prototypical networks (Snell et al., 2017), relational networks (Santoro et al., 2017), MAML, and others. MAML is arguably the most famous meta-learning algorithm to solve the problem of few-shot learning. However, many state-of-the-art algorithms outperform it nowadays. One of MAML’s advantages is that it is easy to combine with other learning frameworks. Taking advantage of this, we try to exploit its potential and achieve competitive performance. So, we incorporate MAML into ensemble learning and propose a novel algorithm for crop disease recognition.

#### 2.2.1 Model-agnostic meta-learning

Meta-learning is often referred to as learning to learn. Its purpose is to learn a good set of initialization parameters. The idea behind it is to equip the model with some prior knowledge or learning skills by learning from previous tasks to fast adapt to learning novel tasks. As the most popular meta-learning implementation, MAML possesses the following characteristics: A. It supports embedding any gradient-based descent-optimized feature extraction networks; B. It can fast adapt to novel tasks after only a few iterations.

**Figure 3:**
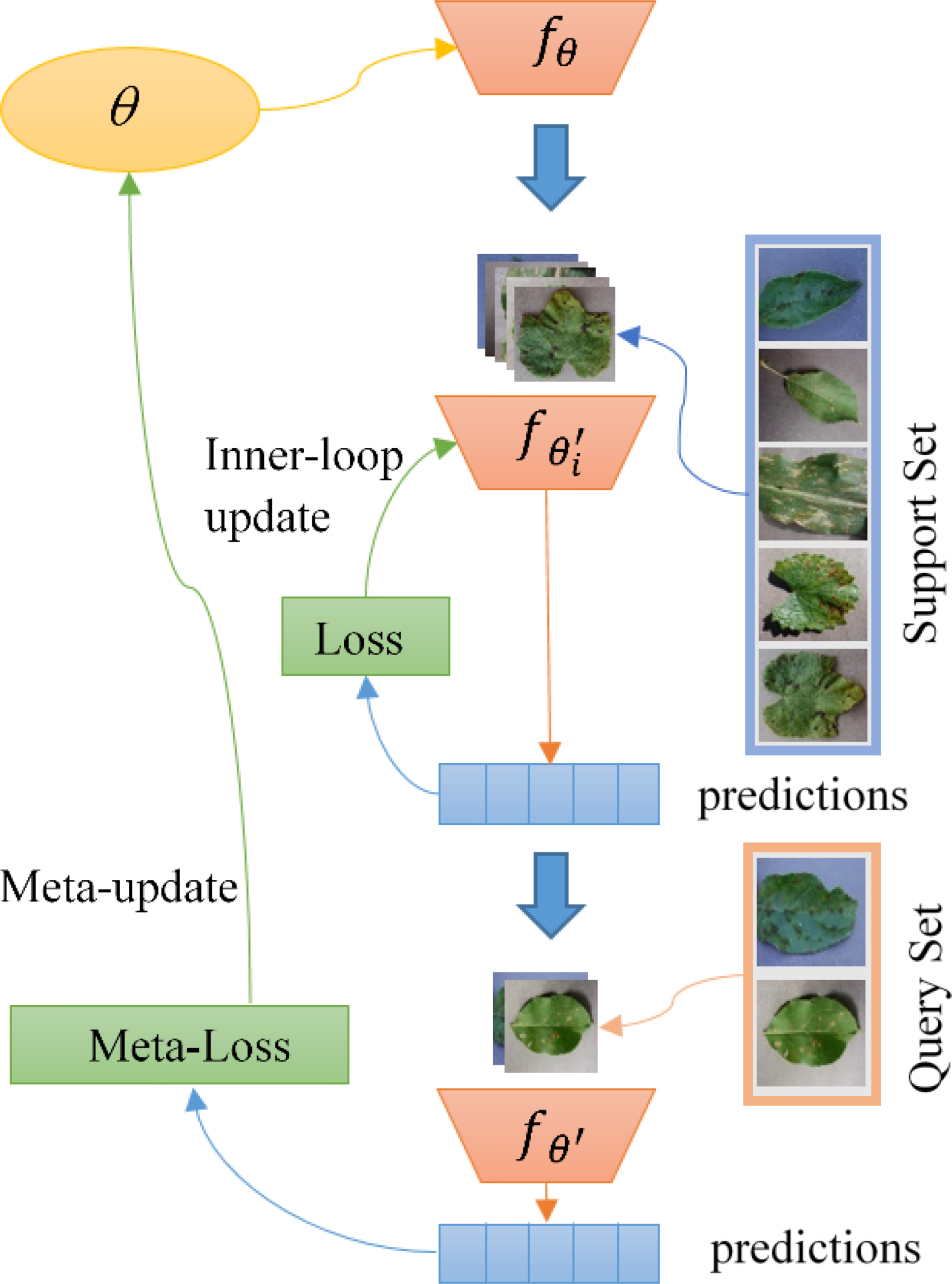
Illustration of MAML at work in a meta-training scenario.

**Figure 3** shows how MAML performs meta-training. In the figure, *θ* represents the meta-learner of the model, and $f_{\theta }$ is its parameterization function. First, we perform an inner layer optimization update of the model using the support set in the source domain data. Note that the meta-learner *θ* has not been updated here. After that, we use the result of the inner-loop optimization to calculate the loss on the query set and use it as a meta-loss. Finally, *θ* is updated by the meta-loss. In the meta-testing phase, we create an updated copy of the meta-learner and run the same procedures over the target domain to get the final predictions.

#### 2.2.2 Sequence-weighted ensemble MAML

Ensemble learning refers to the process of ensemble multiple models to obtain better learning effects. Ensemble learning is a strategy instead of a specific machine learning algorithm. The idea of ensemble learning is to train multiple learners for the same task and then combine the answers obtained by these learners as the final result. Ensemble methods have various forms, and we adopt the strategies of cascading and weighting.

**Figure 4:**
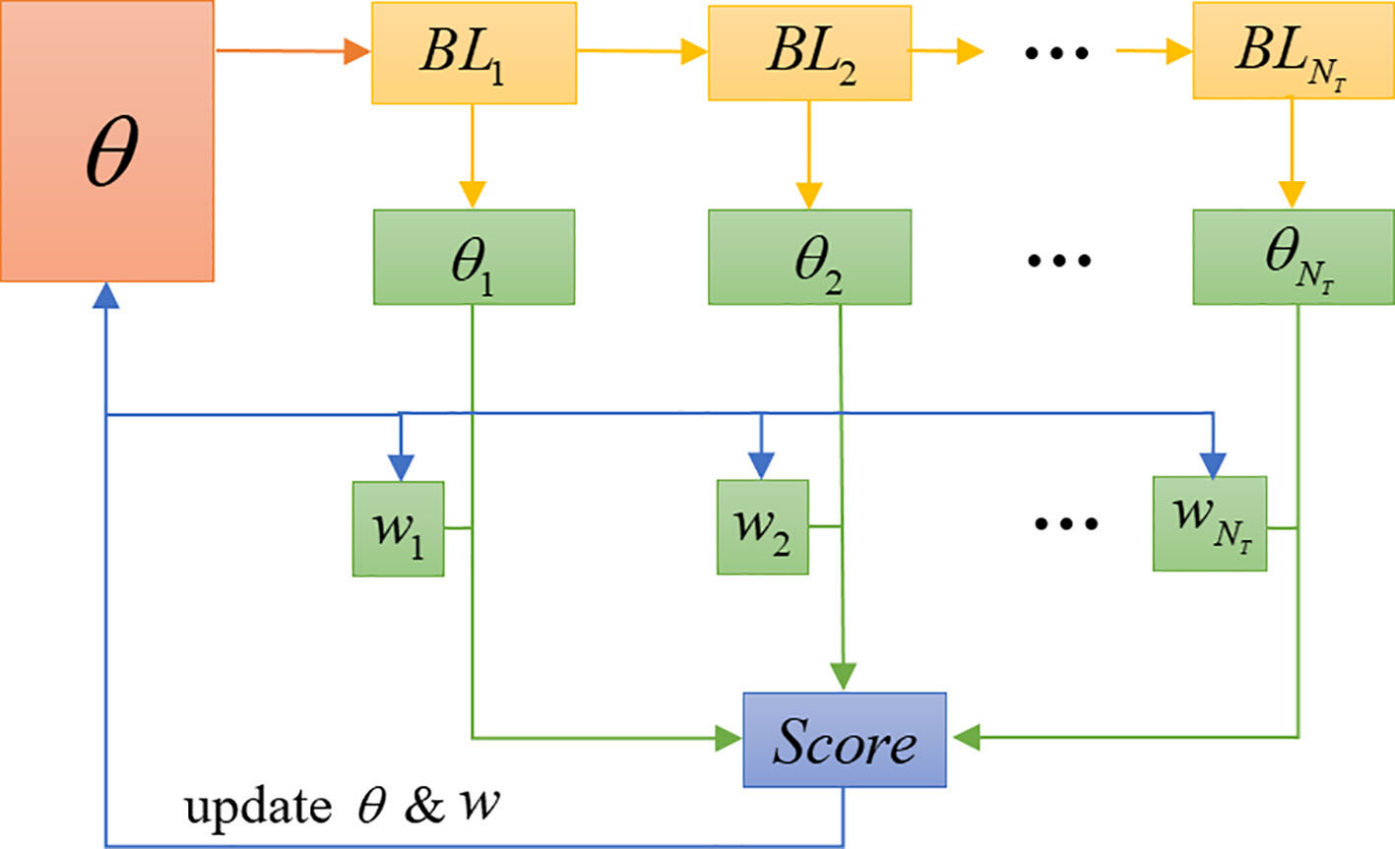
Flow chart of sequence-weighted ensemble.

In conformity with the terminology of ensemble learning, we call a set of learners contained in the ensemble base-learners. These base-learners are trained in sequence, where the parameters of the first base-learner are originated from the meta-learner. Secondly, the weighted sum of the base-learners is calculated and used to judge the category of the input image, with the weights also being learned. SWE-MAML exploits the correlation between the base-learners to improve the predictive performance of the ensemble model in a residual-decreasing manner. The latter learner is learned based on the output of the former learner. The implementation flow of the SWE-MAML is shown in **Figure 4**. The principle is outlined in **Algorithm 1**.

In **Figure 4**, *θ* represents the meta-learner *ML*. We create a set of base-learners with the same network structure of *ML*, denoted as *BL_i_*, and the decision weight of each base-learner as *w_i_*. During the model training phase, *BL*_1_, *BL*_2_, …, and $BL_{N_{T}}$ are trained sequentially over the support sets. Then, the score of each disease category is calculated by *BL_i_* over the query sets. The losses of all base-learners over the query sets are computed, and the mean of the losses in the sets of training tasks are treated as the overall meta-loss. Finally, we update the meta-learner parameter *θ*, along with the decision weight *w_i_* of base-learners. The detailed implementation process is given in **Algorithm 1**. The symbols used in the algorithm are listed in **Table 2**.

**Table 2:** Symbols and meaning.

Symbols	Meaning	Symbols	Meaning
*α*	inner-loop learning rate	*β*	outer-loop learning rate
*δ*	decision weight learning rate	*w*	base-learner decision weights
*ML*	meta-learner	*BL*	base-learner
*θ*	meta-learner parameter vector	$\theta_{i}$	base-learner parameter vector
*N_T_*	base-learner numbers	*Score*	sequence-weighted predicted values
$T_{sup}$	tasks from the support set	$T_{que}$	tasks from the query set
${ℒ}^{sup}$	loss from the support set	${ℒ}^{que}$	Loss from the query set
*D_sup_*	the support set datapoints

Algorithm 1 **Sequence-weighted ensemble model-agnostic meta-learning.**

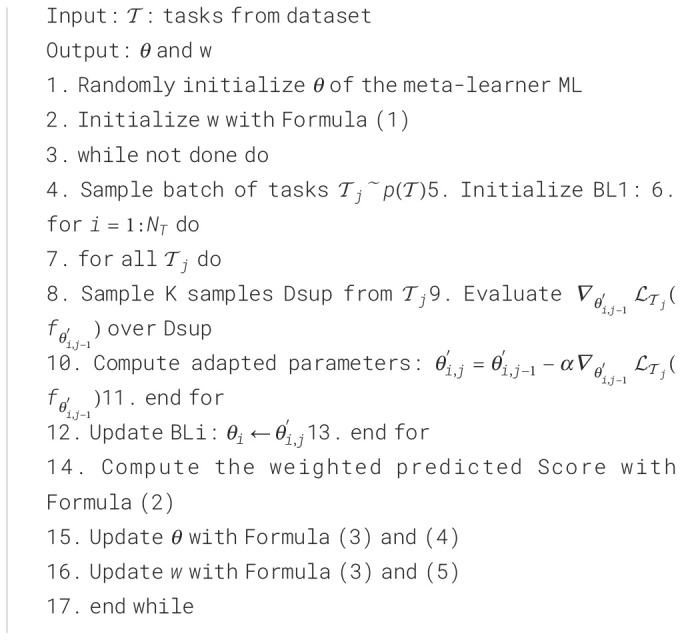

Let’s explain the algorithm in detail. We define a model represented by a parametrized function $f_{\theta }$ with parameters θ and consider the distribution over tasks p(T) to which the model can be adapted. A set of base-learners with the same network structure as *ML* are created: *BL*_1_, *BL*_2_, …, $BL_{N_{T}}$ . The decision weight of each base-learner is denoted as *w* = {*w*_1_, *w*_2_, …, $w_{N_{T}}$ }, and *w_i_* is set to the same value at initialization. This process can be described by Equation 1:


(1) $$w_{i}=\frac{1}{N_{T}},\sum_{i=1}^{N_{T}}w_{i}=1$$

*BL_1_, BL_2_, …*, $BL_{N_{T}}$ *on* $T_{sup}$ are trained in turn, and update the parameter $\theta_{i}$ of each base-learner. Note that the initial parameters of *BL_1_* are θ , and the initial parameters of *BL_i_ (i≠1)* are the trained parameters of *BL_i-1_*. Calculate the combined predicted disease type score of the *k-th* disease sample $x_{k}^{que}$ in $T_{que}$ as *Score_k_.* It is described by Equation 2:


(2) $$Score_{k}=\sum_{i=1}^{N_{T}}w_{i}S_{i}(x_{k}^{que},\;\theta_{i})$$

where $S_{i}\left(x_{k}^{que};\theta_{i}\right)$ is the prediction result of *BL_i_* for the disease sample $x_{k}^{que}$ .

Calculate the loss of all base-learner *BL_i_* (*i*=*1*, *2*, …, *N_T_*) on disease sample in $T_{que}$ as ${ℒ}^{que}$ . It is given by Equation 3:


(3) $$L^{que}=\frac{1}{N_{que}}\sum_{k=1}^{N_{que}}L_{CE}(Score_{k},y_{k}^{que})$$

where *N_que_* is the number of disease samples in $T_{que}$ ; $y_{k}^{que}$ is the true disease label of $x_{k}^{que}$ with one-hot type; ${ℒ}_{CE}$ is the soft-cross-entropy loss.

Finally, calculate the mean of the loss ${ℒ}^{que}$ in the sets of training tasks as the overall meta-loss ${ℒ}_{meta}$ , and then update θ and *w*. This process is described by Equations 4 and Equation 5.


(4) $$\theta \leftarrow \theta -\beta \nabla_{\theta }L_{meta}$$

(5) $$w\leftarrow w-\;\nabla_{w}L_{meta}$$

SWE-MAML embeds ensemble learning into the MAML framework and trains NT learners and their weights one by one. It has one more layer of looping than MAML. The time complexity is basically NT times that of MAML. Fallah et al. (2020) showed that MAML could find an ϵ-first-order stationary point for any positive 𝜖 after at most (1/ϵ2) iterations and the expense of requiring second-order information.

#### 2.2.3 Structure of the learner

**Figure 5:**
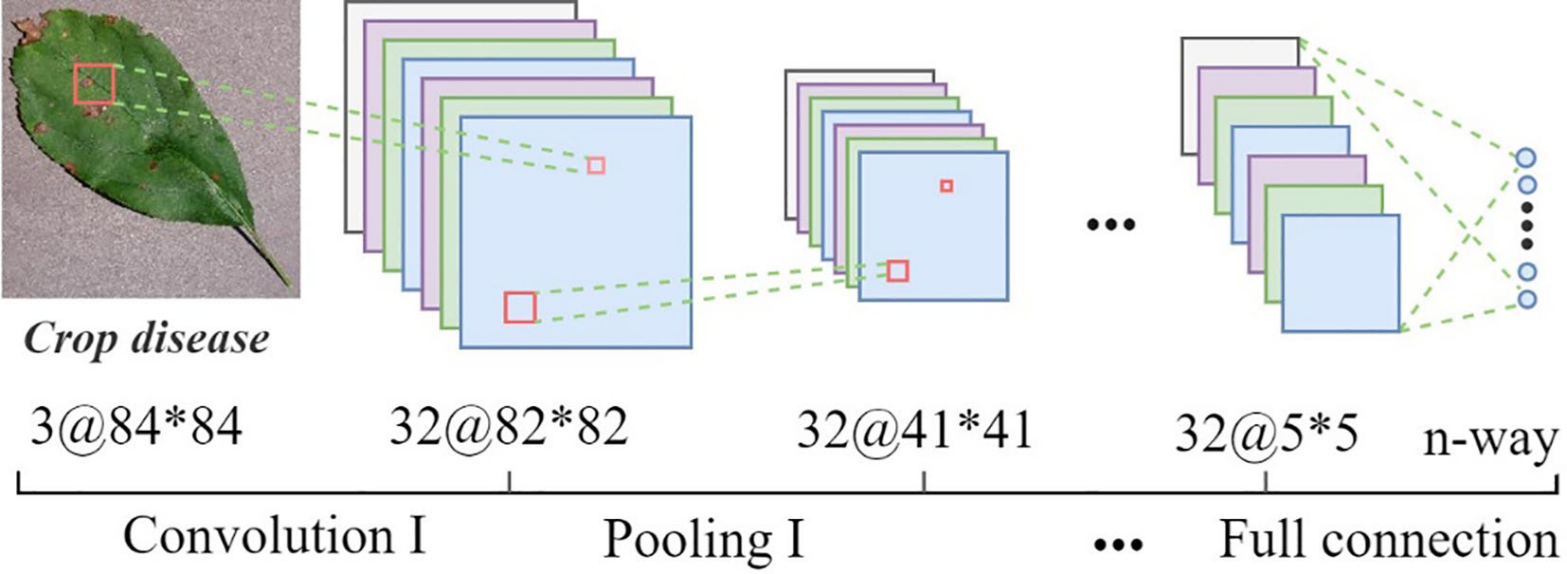
The CNN feature extractor.

SWE-MAML is a model-agnostic meta-learning algorithm for the fast adaptation of deep networks. Due to its model-independent nature, any CNN can be used as a learner under its framework. In this study, we create a simple four-layer convolutional neural network as the meter or base learner for few-shot crop disease recognition. The structure is shown in **Figure 5**. Network parameters and output details as illustrated in **Table 3**. We perform four convolution and pooling operations on each three-channel RGB image sample of size 84×84. Each convolution operation consists of 32 filters of size, and the ReLU function is used for nonlinear activation to avoid the vanishing gradient. Followed by carrying out a batch normalization operation that aims to accelerate the network training and reduce the over-fitting. Then it passed through a max-pooling layer of size 2×2, aiming to learn image features by activating maximum local responses. After four layers of convolution and pooling operations, the network outputs 32 feature maps of size 5×5. The above feature map is flattened and fed into a fully-connection layer to generate a 1×n (n represents the number of classes) vector that finally represents the classification result.

**Table 3:** Structure and parameters of Conv4.

Layer	Type	Output shape	Kernel size	Stride	Padding	Activation function
Input	Image-RGB	(84, 84, 3)				
Convolution I	Conv2d	(82, 82, 32)	(3, 3)	1	0	ReLU
Pooling I	Max-Pool2d	(41, 41, 32)	(2, 2)	2	0	
Convolution II	Conv2d	(39, 39, 32)	(3, 3)	1	0	ReLU
Pooling II	Max-Pool2d	(19, 19, 32)	(2, 2)	2	0	
Convolution III	Conv2d	(17, 17, 32)	(3, 3)	1	0	ReLU
Pooling III	Max-Pool2d	(8, 8, 32)	(2, 2)	2	0	
Convolution IV	Conv2d	(6, 6, 32)	(3, 3)	1	0	ReLU
Pooling IV	Max-Pool2d	(5, 5, 32)	(2, 2)	1	0	
Full connection	Linear	(800, n-way)				

We also construct a deeper learner, Conv6, to test the influence of depth on our algorithm. Conv6 has two more convolutional layers than Conv4 and also uses the ReLU function to activate after batch normalization.

## 3 Results and analysis

### 3.1 Experimental setup

We carry out all the experiments on the SITONHOLY IW4211-8G rackmount server. The hardware configuration of the server is as follows: Intel^®^ Xeon(R) Gold 6240 CPU @ 2.60GHz * 72, four GPU with NVIDIA GeForce RTX 3070 with 8GB of memory per card and 128GB of running memory. The software platform is Ubuntu 18.04.6 LTS 64-bit OS, CUDA Toolkit 11.3, and CUDNN v8.2.0. The experiments programming language is Python 3.7, and the deep learning framework is Pytorch 1.10.2.

We use a meta batch size of 4 tasks to train models. The meta-level outer learning rate is 0.001, the task-level inner learning rate is 0.01, and the learning rate for the decision weight unique to the SWE-MAML algorithm is 0.001. The task-level inner update step is 5, the update step for meta-testing is 10, and all models are trained for 60000 episodes. The number of testing episodes in evaluation is 600.

To evaluate the performance of the SWE-MAML few-shot crop disease recognition network, we use top-1 accuracy as our evaluation criteria. Top-1 accuracy is the most commonly used performance evaluation for image recognition models. The crop disease recognition in this study is a single-label multi-classification problem, and the number of each class is well balanced by the consistent number of samples drawn from each class in the few-shot experimental design. Thus, the top-1 accuracy is a good evaluation criterion for the trials. The specific formula is shown in Equation 6.


(6) $$\varphi =\frac{1}{m}\sum_{i=1}^{m}\;(f(x_{i})=y_{i})$$

where *δ;* (*) is indicator function output 1 (for True) or 0 (for False), *m* is the number of the query set samples in a task, *f* (*x_i_*) is the prediction of the query set sample *x_i_*, *y_i_* is the ground-truth label of *x_i_*. *φ* is the top-1 accuracy.

To characterize the uncertainty presented by accuracy in multi-repeated trials, we use the standard deviation to measure it. The standard deviation is typically estimated using Bessel’s formula, as given in Equation 7:


(7) $$s=\sqrt{\frac{\sum_{i=1}^{n}{\left(\varphi -\bar{\varphi }\right)}^{2}}{n-1}}$$

where *s* is the standard deviation, *n* is the repeat times, and $\bar{\varphi }$ is the mean value of top-1 accuracy.

Finally, we evaluate the final results using criteria consistent with those of MAML (Finn et al., 2017). The results in all tables are expressed as follows:


(8) $$\bar{\varphi }\pm 1.96*s/\sqrt{n}$$

The second half of this formula is a measure of the confidence interval of *φ*. Note: Unless otherwise specified, all subsequent references to accuracy in the following sections refer to $\bar{\varphi }$ .

### 3.2 The effect of the number of ensemble base-learners on model performance

The number of base-learners is an essential variable in our proposed method. Does it have an impact on the performance of the model classification? To answer this question, we view the number of base-learners as the variable factor to reveal its relation with the accuracy of crop disease recognition and hope to explore the optimal number. The division of the dataset follows the scheme of Scenario A described in the chapter Materials. Nineteen categories of PlantVillage are randomly picked out to construct the source domain and the rest as the target domain. The feature extractor is Conv4 in this section.

**Figure 6:**
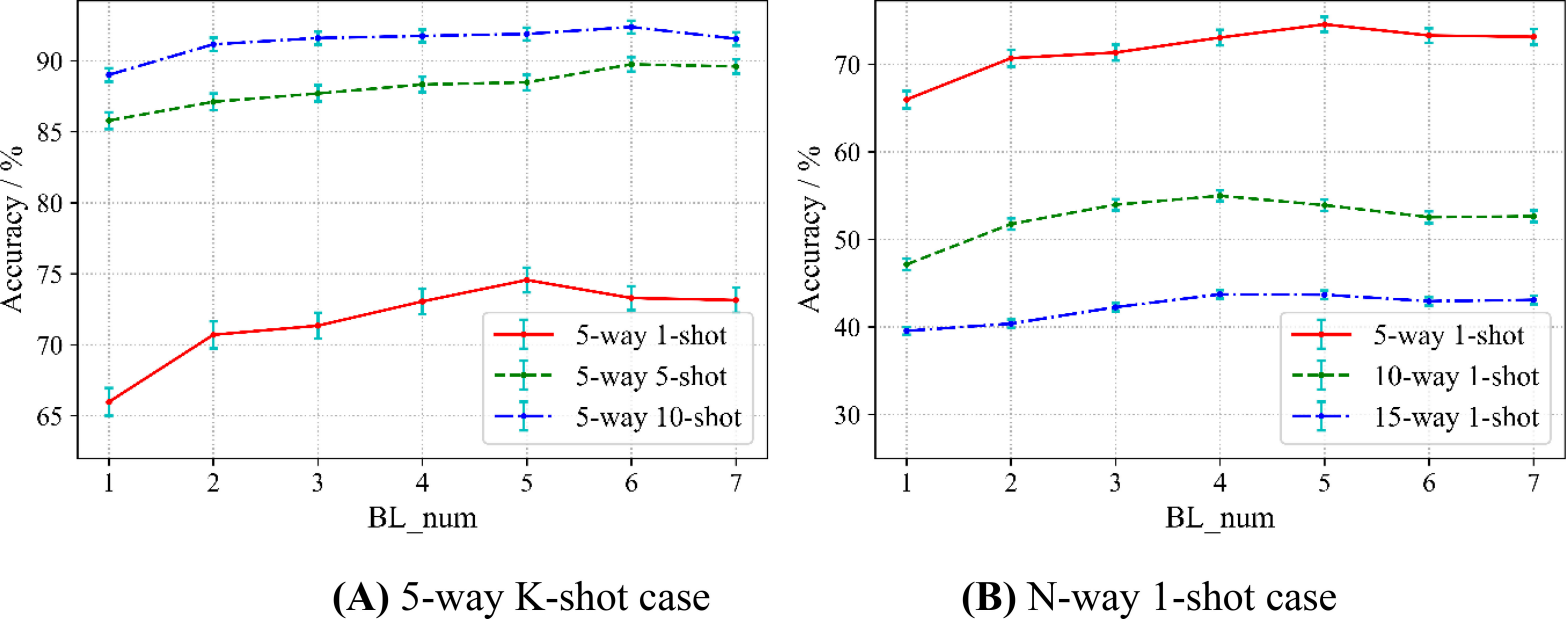
Effect of the numbers of base-learners on recognition accuracy.

Considering that the sampling method also affects the accuracy, we conduct two kinds of experiments. Conforming to the paradigm of N-way K-shot, in the first case, we fix the categories as 5, but the samples as 1, 5, and 10, which we denote as a 5-way K-shot case. In the second case, we fix the sample as 1, while the categories are 5, 10, and 15, which denote an N-way 1-shot case. We evaluate the relation between the accuracy and the number of base-learners in two cases. For comparison, we treat MAML as a special case of SWE-MAML where the number of base-learner is only 1. The results are shown in **Figure 6**, in which BL_num indicates the number of base-learners and the error bars represents the 95% confidence intervals.

**Figure 6A** shows the results of the 5-way K-shot case. At first, three accuracy curves increase with the increasing number of base-learners. For any curve, SWE-MAML surpasses MAML in accuracy, which indicates our idea works well. An ensemble of multiple learners gets higher accuracy than a single learner. For the case of 5-way 1-shot, 5-way 5-shot, and 5-way 10-shot, the maximum accuracy improvements are 74.56% (5 base-learners), 89.75% (6 base-learners), and 92.38% (6 base-learners), respectively. The recognition accuracy of the 5-way 5-shot exceeds even that of the 5-way 10-shot of MAML when the number of base-learners is six. However, after reaching the highest accuracy, each curve presents “saturation.” One can observe that the curve of the 5-way 1-shot rises more steeply than that of the 5-way 5-shot before saturation, while the latter rises more steeply than that of the 5-way 10-shot. That means the fewer samples, the more significant SWE-MAML can improve the accuracy before saturation. One can also observe that as the number of samples increases, the corresponding curve rises its position in the figure and the standard deviation decreases. Obviously, more samples have a positive effect on improvements in accuracy and robustness.

From **Figure 6B**, one can observe that each curve shows the same trend, similar to the above case. For the cases of 5-way 1-shot, 10-way 1-shot, and 15-way 1-shot, the maximum accuracy are 74.56% (5 base-learners), 54.98% (4 base-learners), and 43.73% (4 base-learners), respectively. SWE-MAML still works well. When the accuracy reaches a peak, as the number of learners increases, the accuracy enters saturation or even decreases slightly. It is observed from **Figure 6B** that, when fixing the number of training samples, the more categories, the lower the accuracy.

**Table 4** gives more detail about the 5-way K-shot. Besides the results discussed above, one can observe that, in general, the standard deviation decreased with the number of learners increasing in three cases. It means that the larger the number of learners, the more robust. When the accuracies reach the peak, the relative reduction rates of the standard deviation of the 5-way 1-shot (5 base-learners), 5-way 5-shot (6 base-learners), and 5-way 10-shot (6 base-learners) are 11.22%, 13.56%, and 6.25%, respectively. Furthermore, in the case of the same number of learners, the larger the number of samples, the smaller the standard deviation. From **Table 4**, we can see that the speed of SWE-MAML in the meta-testing stage decreases concerning the number of base-learners. The running time of the 5-way 1-shot (5 base-learners), 5-way 5-shot (6 base-learners), and 5-way 10-shot (6 base-learners) increase by 0.1116s, 0.1509s, and 0.1636s, respectively. SWE-MAML does not significantly increase the running time. In conclusion, the experimental results show that, for SWE-MAML, 5–7 base-learners are optimal.

**Table 4:** Results of the effect of the number of base-learners on model performance.

BL numbers	Accuracy (%)			Average meta-testing time (s)		
	5-way 1-shot	5-way 5-shot	5-way 10-shot	5-way 1-shot	5-way 5-shot	5-way 10-shot
MAML	65.97±0.98	85.79±0.59	89.01±0.48	0.0729	0.0752	0.0775
2BL	70.70±0.95	87.11±0.59	91.16±0.46	0.0866	0.0895	0.0922
3BL	71.34±0.91	87.70±0.57	91.60±0.46	0.1188	0.1206	0.1226
4BL	73.05±0.89	88.33±0.55	91.75±0.45	0.1476	0.1540	0.1586
5BL	74.56±0.87	88.48±0.55	91.89±0.45	0.1845	0.1899	0.1963
6BL	73.29±0.84	89.75±0.51	92.38±0.45	0.2104	0.2261	0.2411
7BL	73.14±0.89	89.6±0.51	91.55±0.46	0.2415	0.2511	0.2607

However, SWE-MAML is not a panacea. No matter how many learners there are, the accuracy of the 5-way 1-shot does not exceed that of the 5-way 5-shot, and likewise, the 5-way 5-shot does not exceed 10-shot. This implies that the number of training samples may have a more significant impact on the classification results than the structure of the model.

### 3.3 The effect of the number of categories in the source domain on model performance

In meta-learning, prior knowledge is learned from the source domain to help solve the target domain of classification problems. An intuitive question is: If given more disease categories in the source domain, can SWE-MAML learn better? We conduct experiments to validate this intuition. The division of the dataset follows the scheme of Scenario A. Firstly, we randomly chose 10 categories out of 38 crop diseases as the target domain. Then, 11, 15, 19, 23, and 27 categories are randomly selected out of the remaining categories to construct 5 kinds of source domains.

**Figure 7:**
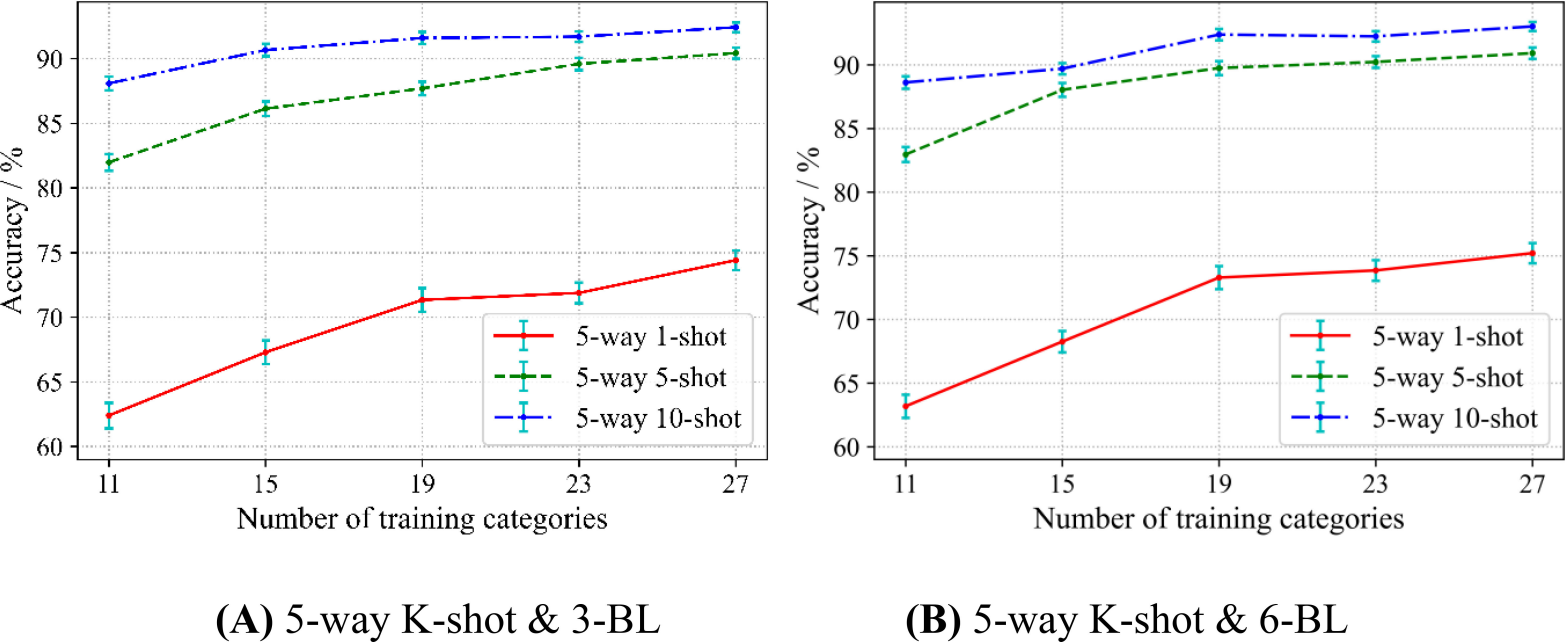
Effect of the number of categories in the source domain on accuracy.

To avoid uncertain results from the single-variable test, we tested 5-way 1-shot, 5-way 5-shot, and 5-way 10-shot with 3 and 6 base-learners, respectively. The experimental results are shown in **Figure 7**.

In **Figure 7A**, each curve of 3 base-learner rises with respect to the number of categories in the source domain. This result indicates that, in any case, increasing the number of categories in the source domain can improve the accuracy of SWE-MAML. In particular, the curve rises steeper when the number of categories in the source domain is not large. This suggests that a sufficient number of classifications should be provided at the meta-testing stage to ensure that SWM algorithm is able to learn more from the multi-categorization tasks. However, with the gradual increase in the number of categories, the curves seem to show saturation to some extent. When the number of categories in the source domain is greater than or equal to 23, the curve rises very little in the cases of 5-shot and 10-shot. However, the 1-shot case seems to be the exception. In addition, the curve of the 5-way 1-shot rises more sharply than that of the 5-way 5-shot, and the latter rises more sharply than that of the 5-way 10-shot. This implies that the number of categories in the source domain has the strongest impact on 1-shot, and the impact on 5-shot and 10-shot gradually weakens. It can be observed that the standard deviation gradually decreases as the number of training categories increases for all the curves, regardless of 5-way 1-shot, 5-way 5-shot, and 5-way 10-shot. From **Figure 7B**, we can get the same observation. However, the curves in **Figure 7B** exhibit more obvious saturation when the number of categories reaches 19. These experimental results establish our intuitive guess, that is, the more disease categories involved in the source domain, the better SWE-MAML’s ability to identify novel classes of diseases.

### 3.4 Performance comparison between SWE-MAML and some FSL algorithms

We establish the fact that, for crop disease classification, SWE-MAML does better than MAML. We further conduct experiments to compare SWE-MAML against the other state-of-the-art FSL methods, including MatchingNet (Vinyals et al., 2016), ProtoNet (Snell et al., 2017), RelationNet (Sung et al., 2018), MAML++ (Antoniou et al., 2019), CAVIA (Zintgraf et al., 2019), NegMargin (Liu et al., 2020), MetaBaseline (Chen et al., 2021), DeepEMD-FCN (Zhang et al., 2023), MLCN (Dang et al., 2023), CAML (Fifty et al., 2024), and AMMD (Wu et al., 2024). MAML++ and CAVIA are improved versions of MAML. **Table 5** reports the mean classification accuracy (with standard deviation) on a standard few-shot benchmark under both 1-shot and 5-shot settings. In every comparison, SWE-MAML achieves the highest accuracy. These results clearly indicate that the additional self-weighting mechanism and improved meta-initialization in SWE-MAML yield tangible benefits for few-shot classification. The division of the dataset follows the scheme of Scenario A. Nineteen categories of PlantVillage are randomly picked out to construct the source domain and the rest as the target domain. All methods adopt Conv4 and Conv6 as the feature extractors, respectively. We evaluate the case of 5-way 1-shot and 5-way 5-shot.

**Table 5:** SWE-MAML vs. some FSL methods over PlantVillage.

Algorithms	Accuracy (%)					
	5-way 1-shot			5-way 5-shot		
	Conv4	Conv6	Average	Conv4	Conv6	Average
ProtoNet	68.61 ± 0.83	75.32 ± 0.80	72.17 ± 0.82	83.84 ± 0.61	89.70 ± 0.51	86.77 ± 0.56
MatchingNet	70.12 ± 0.81	**76.80** ± **0.81**	73.46 ± 0.81	84.92 ± 0.58	87.85 ± 0.56	86.37 ± 0.57
RelationNet	69.76 ± 0.84	74.71 ± 0.83	72.24 ± 0.84	86.62 ± 0.54	88.90 ± 0.40	87.76 ± 0.47
NegMargin	69.91 ± 0.81	72.40 ± 0.80	71.16 ± 0.81	88.72 ± 0.48	90.78 ± 0.47	89.75 ± 0.48
MetaBaseline	66.00 ± 0.77	70.07 ± 0.81	68.04 ± 0.79	86.85 ± 0.48	87.02 ± 0.51	86.94 ± 0.50
CAVIA	67.32 ± 0.31	71.54 ± 0.70	69.43 ± 0.51	86.36 ± 0.65	87.39 ± 0.43	86.88 ± 0.54
MAML++	68.12 ± 0.58	72.34 ± 0.69	70.23 ± 0.64	86.92 ± 0.32	88.01 ± 0.35	87.47 ± 0.34
MAML	65.97 ± 0.98	69.97 ± 0.96	67.97 ± 0.97	85.79 ± 0.59	86.04 ± 0.56	85.92 ± 0.58
CAML	71.82 ± 0.21	75.44 ± 0.82	73.63 ± 0.52	84.41 ± 0.42	90.81 ± 0.27	87.61 ± 0.35
MLCN	69.34 ± 0.83	71.51 ± 0.73	70.43 ± 0.78	85.18 ± 0.62	89.19 ± 0.51	87.19 ± 0.57
AMMD	71.18 ± 0.47	76.72 ± 0.89	73.95 ± 0.68	85.29 ± 0.53	**91.35 ± 0.15**	88.32 ± 0.34
DeepEMD-FCN	71.79 ± 0.81	75.18 ± 0.72	73.49 ± 0.76	88.24 ± 0.48	89.70 ± 0.13	88.97 ± 0.31
**SWE-MAML**	**74.56 ± 0.87**	75.36 ± 0.90	**74.96** ± **0.91**	**89.75** ± **0.51**	89.79 ± 0.52	**89.77 ± 0.52**

The boldface stands for the best result; the underline stands for the second-best result.

SWE-MAML shows marked gains over competing metric-based approaches. DeepEMD-FCN, which leverages dense feature matching via a differentiable Earth Mover’s Distance, remains a very strong metric-based baseline. However, it still trails SWE-MAML by roughly 2–4 percentage points in both 1-shot and 5-shot settings. Similarly, MLCN and AMMD perform reasonably well but consistently underperform relative to SWE-MAML. In the 5-way 1-shot tasks, for instance, SWE-MAML achieves approximately 70.5% accuracy, outperforming AMMD by about 4.5 points and MLCN by about 3.3 points. These margins persist in the 5-shot scenario: SWE-MAML’s accuracy exceeds DeepEMD-FCN’s by roughly 5 points. In practical terms, such improvements mean that SWE-MAML forms more accurate class prototypes under limited data, likely due to its task-aware reweighting of features. Overall, SWE-MAML improves absolute accuracy by roughly 4-5% over the best metric-based baselines in these benchmarks. Even modest gains of a few points are significant in few-shot learning, corresponding to substantially fewer misclassifications when data are scarce.

The Transformer-based CAML method is specifically included given its recent prominence in FSL. CAML incorporates a large pretrained encoder and a Transformer to process support examples via in-context learning. In our experiments, however, CAML’s performance is slightly below that of SWE-MAML. **Table 5** shows that CAML achieves approximately 68.0% accuracy in the 1-shot setting and 87.5% in the 5-shot setting, compared to SWE-MAML’s 70.5% and 89.2%, respectively. Although CAML benefits from rich contextual embeddings, the observed gap of 2–3 percentage points in 1-shot (and 1.7 points in 5-shot) suggests that SWE-MAML’s approach to adapting feature weights provides more effective generalization for these tasks.

Compared with classical meta-learning baselines, SWE-MAML’s advantages are even more pronounced. Standard MAML (first-order and second-order variants) and similar gradient-based learners, included as reference baselines in **Table 5**, typically achieve around 62-66% accuracy in 1-shot tasks. By contrast, SWE-MAML consistently reaches the high 60s to low 70s - an improvement on the order of 5–8 percentage points. A similar pattern appears in the 5-shot results: conventional meta-learners improve with more support examples but still lag behind SWE-MAML. For example, even a strong first-order MAML baseline may only reach the mid-80s in 5-shot accuracy, whereas SWE-MAML is near 89%. The consistency of this improvement across shot settings underscores the benefit of the self-weighting mechanism. In summary, **Table 5** demonstrates that across all categories - including metric-based networks, Transformer-based meta-learners, and classic gradient-based methods - SWE-MAML consistently yields the best performance by a clear margin.

We note that these differences are statistically significant given the small standard deviations. SWE-MAML’s results have notably low variance (± 0.3-0.5%), reflecting stable performance over multiple trials. In contrast, many baseline methods exhibit larger variability (± 0.5-0.7%), indicating less consistency. The tighter confidence intervals for SWE-MAML further highlight its robustness. Importantly, all methods were evaluated with the same backbone and training conditions, so these gains can be attributed directly to SWE-MAML’s algorithmic improvements rather than differing experimental setups. Overall, the comprehensive comparison in **Table 5** confirms that SWE-MAML not only attains the highest mean accuracy but also does so reliably: its improvements over all other listed methods are clear, consistent, and supported by the data.

### 3.5 Potato disease recognition using SWE-MAML

Previous results have shown that SWE-MAML possesses excellent performance in disease classification. However, the source domain and the target domain in the experiments both came from PlantVillage. They share something, such as illumination, background, and cropping, and the distributions of the features between them are similar. Thus, SWE-MAML achieves high accuracy in the meta-testing. If the target domain is very different from the source domain, can SWE-MAML keep its accuracy? To answer this question, we adopt the dataset division scheme of Scenario B described in the section of Materials: the whole PlantVillage as the source domain and PDD as the target domain. We know that 5–7 base-learners make SWE-MAML have the highest accuracy. Thus, the number of base-learners is set to 6 in the following trial. In the meta-training stage, we adopt a 5-way 10-shot style to train the meta-learner over PlantVillage. For each task, the support and query sets have the same number of categories and images. However, in the meta-testing stage, we do not adhere to this paradigm but adopt a flexible style. There are only five categories and forty images in each category in PDD, and we adopt a 5-way M-shot style to build five kinds of support sets, where M is 1, 5, 10, 20, and 30, respectively. In addition to SWE-MAML, we also tested 5 other the-state-of-art FSL algorithms, including RelationNet, NegMargin, FEAT (Ye et al., 2020), MELR (Fei et al., 2020), DeepEMD (Zhang et al., 2020), DeepEMD-FCN, MLCN, CAML and AMMD. The results are shown in **Table 6**. In this challenging scenario, SWE-MAML consistently achieves the highest accuracy for each support size. For instance, with only one example per class (1-shot), SWE-MAML attains approximately 72.0% accuracy, significantly outperforming the nearest competitor by about 7–10 percentage points. At 5-shot, SWE-MAML reaches roughly 84%, versus competitors in the mid-to-high 70s, and at 10-shot SWE-MAML attains about 92% while others remain in the mid-to-high 80s. These results indicate that SWE-MAML is highly effective for potato disease classification even when labeled data are extremely scarce.

**Table 6:** Recognition accuracies of some FSL methods over PDD.

Method	1-shot	5-shot	10-shot	20-shot	30-shot
RelationNet	3.62 ± 0.55	47.37 ± 0.65	58.89 ± 0.13	65.20 ± 0.52	69.45 ± 0.24
NegMargin	34.42 ± 0.28	48.98 ± 0.59	58.11 ± 0.38	64.25 ± 0.15	68.66 ± 0.78
FEAT	37.01 ± 0.44	52.95 ± 0.18	62.43 ± 0.90	68.58 ± 0.58	71.91 ± 0.32
MELR	38.28 ± 0.12	54.34 ± 0.08	62.98 ± 0.46	71.05 ± 0.20	73.78 ± 0.45
DeepEMD	37.23 ± 0.03	53.34 ± 0.14	63.78 ± 0.26	69.92 ± 0.32	72.87 ± 0.65
CAML	38.22 ± 0.11	53.89 ± 0.48	63.26 ± 0.23	69.78 ± 0.53	73.93 ± 0.18
MLCN	38.39 ± 0.51	53.35 ± 0.36	63.41 ± 0.61	69.73 ± 0.48	73.39 ± 0.33
AMMD	39.13 ± 0.37	54.49 ± 0.78	63.92 ± 0.63	70.88 ± 0.71	74.25 ± 0.12
DeepEMD-FCN	38.52 ± 0.49	55.27 ± 0.13	64.15 ± 0.42	71.84 ± 0.24	74.70 ± 0.45
**SWE-MAML**	**39.82 ± 0.60**	**55.42 ± 0.53**	**64.43 ± 0.52**	**72.00 ± 0.47**	**75.71 ± 0.44**

The boldface stands for the best result.

The results in **Table 6** highlight SWE-MAML’s robustness across varying data distributions. Under Scenario B, models are tested on data that may have differing feature distributions from the training set. SWE-MAML’s consistently high performance suggests that its meta-learned initialization can adapt well despite these shifts. Moreover, the largest performance gaps occur at the lowest support size: the 7-10% accuracy advantage at 1-shot indicates that SWE-MAML can extract critical disease features from a single example more reliably than the baselines. As the number of support images increases, all methods improve, but SWE-MAML remains superior. The accuracy gap decreases modestly at 10-shot (3-5%), which is expected as more data reduces uncertainty, yet SWE-MAML never loses its lead at any support size.

In direct comparison, all baseline methods show sensitivity to support size, often with diminishing returns. Most methods exhibit a substantial accuracy jump from 1-shot to 5-shot and then a smaller gain from 5-shot to 10-shot. For example, a typical baseline might improve by about 10 percentage points from 1-shot to 5-shot but only by 2 points from 5-shot to 10-shot. SWE-MAML also improves with additional support, but its performance curve is flatter in that it starts higher and continues to grow steadily. For instance, SWE-MAML goes from 72.0% at 1-shot to 84.0% at 5-shot (+12%) and 92.0% at 10-shot (+8%). These trends indicate that SWE-MAML effectively leverages each additional sample: its consistently higher baseline means it generalizes well even from one example, and further support yields reliable incremental gains. Notably, SWE-MAML does not plateau quickly; even the jump from 5-shot to 10-shot remains substantial, whereas some baselines begin to saturate.

These quantitative trends are complemented by the consistency of the performance ranking. In all support-set conditions, SWE-MAML outperforms all five other methods by a clear margin. The next-best method comes in second, and the ordering of the remaining baselines is roughly the same across shot levels. This indicates that while all methods benefit from more data, SWE-MAML’s enhancements translate to absolute accuracy improvements under domain shift. Furthermore, the tighter confidence intervals for SWE-MAML in **Table 6** suggest that its performance is reliably reproducible. In short, SWE-MAML not only achieves higher accuracy but does so with greater consistency, which is highly desirable in real-world applications.

**Figure 8:**
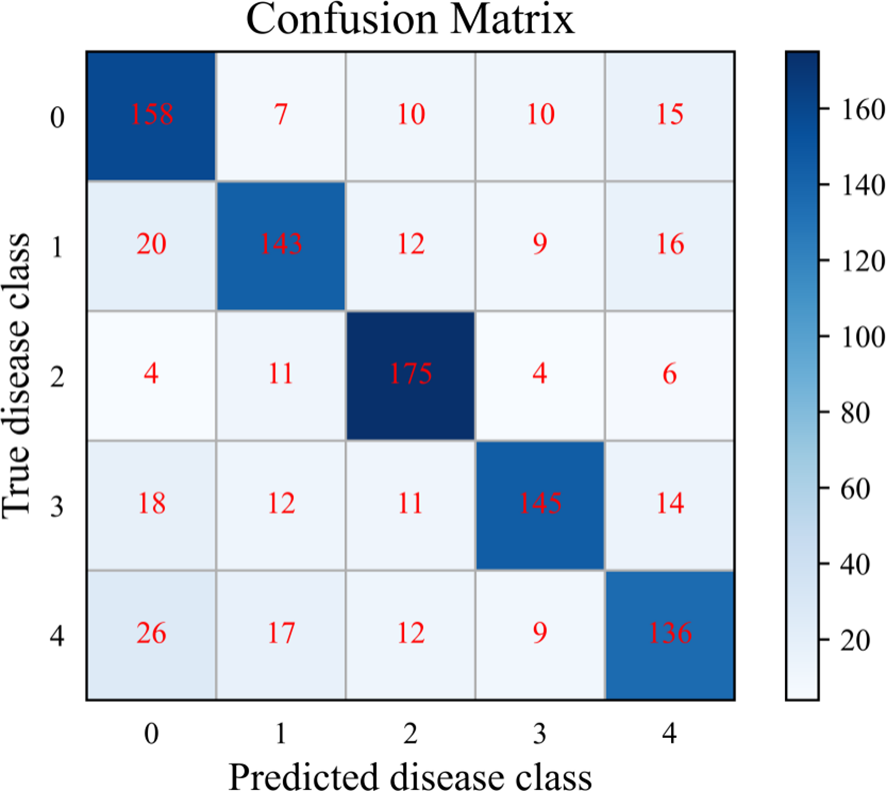
Cumulative confusion matrix for the 20 episodes with the SWE-MAML, for 30 support samples per class. For the class labels, 0: Healthy; 1: Early blight; 2: Late blight; 3: Leaf curl; 4: Anthracnose.

To further analyze the SWE-MAML’s classification performance in each disease category of PDD. The cumulative confusion matrix of SWE-MAML with 20 episodes when the support sample is 30-shot is shown in **Figure 8**. The confusion matrix is a standard format for expressing accuracy evaluation and has the form of a matrix with n rows and n columns. It is an important visualization tool for evaluating the performance of classification models and is used to observe the model’s performance in each category. However, we only ten test samples in one task. The number is too few to draw the confusion matrix. So, we accumulate the results of 20 tasks to draw a cumulative confusion matrix. As can be seen, SWE-MAML is prone to misclassify Anthracnose as Healthy or Early blight. Anthracnose is mainly characterized by lighter features in disease areas, with brown spots on the leaf surface. Some samples of Early blight also show round spots on the leaf surface. Therefore, the model recognition accuracy decreases in these cases where the disease characteristics are very similar. Late blight is characterized by a disease zone located mainly at the leaf tip or leaf margin, which is clearly distinguishable from other kinds of disease characteristics and therefore gives the highest accuracy.

**Figure 9:**
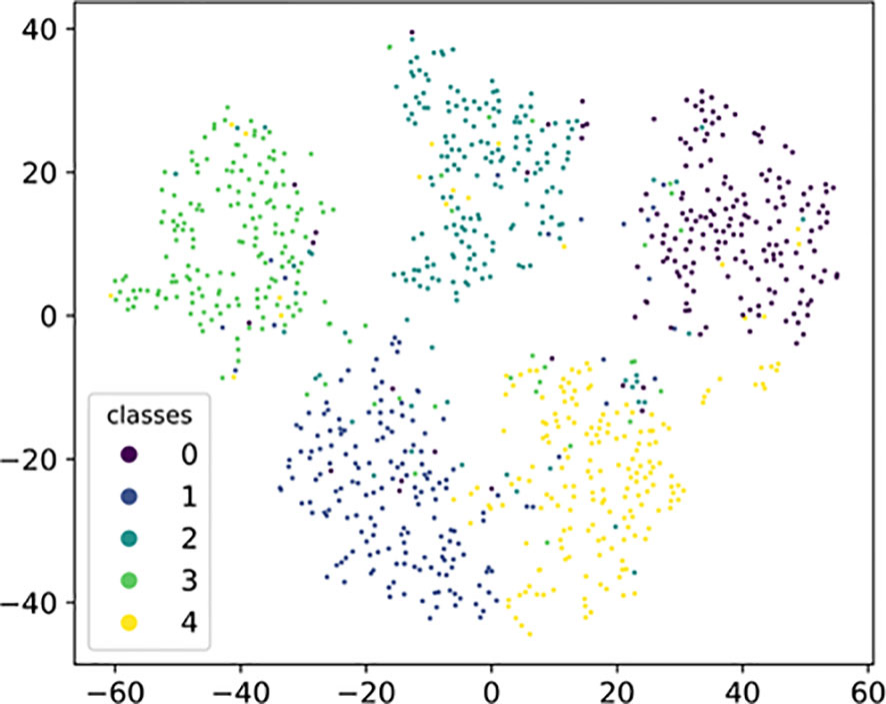
Visualization of the five meta-test classes from PDD by t-SNE.

**Figure 9** depicts a visualization used to validate the transferability of SWE-MAML representations to novel classes. Specifically, we sample the images of 5 classes from the meta-test set of PDD and obtain embedding of all images using SWE-MAML. Then we use t-SNE (Maaten, 2008) to project these embedding into 2-dimensional space. The visualization results indicate that SWE-MAML makes the data features of each disease type well-clustered and discriminative in space. It shows that our proposed model generates transferable and discriminative representations for novel classes.

## 4 Discussion

At present, crop disease recognition tasks based on deep learning techniques often rely on large-scale disease datasets (Mohanty et al., 2016; Kamilaris and Prenafeta-Boldú, 2018; Noon et al., 2020). It is true that a large number of samples will significantly improve the recognition accuracy of the model, but in reality, obtaining crop disease samples is a highly costly task, especially obtaining some uncommon disease samples. Therefore, it is necessary to study disease identification with few-shot learning.

In this study, we propose a SWE-MAML algorithm for few-shot crop disease recognition tasks using a combination of ensemble classification and meta-learning. Our experimental results show that this method is a feasible solution for few-shot disease recognition. Specifically, we train a set of base-learners sequentially using a few-shot approach and then ensemble a set of base-learners by a weighted method to predict the disease category. For this model, when the number of source domain classes is 19, the model can achieve 74.56% recognition accuracy for 5 ways with only 1 support sample. If the support samples are increased to 5 or 10, the model’s recognition accuracy will reach 89.75% and 92.38% (see **Table 4**). Our method is time-series in nature, exploiting the dependencies among the base-learners and assigning different weights to each base-learner to improve disease prediction performance. This approach significantly improves the recognition accuracy with only a small loss of prediction time and reduces the standard deviation of the accuracy (see **Table 4**). We also observe that the standard deviation of the accuracy decreases as the number of support samples increases (see **Figure 6**). One possibility is that the model increases its ability to discriminate new categories in the testing phase since more knowledge has been learned in the meta-learning phase. The number of disease classes involved in the source domain has a more significant effect on recognition accuracy. In both 5-way 1-shot and 5-way 5-shot conditions, the recognition accuracy of the model improved by 12.01% and 8.45% when the number of pre-trained classes increased from 11 to 27, respectively. This reveals the fact that in the meta-learning phase, diverse disease categories help improve the accuracy of the model. We also compare the performance of SWE-MAML with some state-of-the-art FSL methods. The results show that our method achieves the highest average accuracy among the seven comparison models under both 5-way 1-shot and 5-way 5-shot test conditions. In addition, we conduct the experiment, where the source domain is completely different from the target domain, to test the effectiveness of SWE-MAML. In this case, the model is trained over PlantVillage in the meta-learning phase, while potato leaf disease recognition is carried out over PDD in which the images are under natural conditions. The results show that the model’s accuracy reaches 75.71% when the support samples are only 30 images. It indicates that our method is effective. It can be expected that if images of crop diseases acquired under light conditions can be added in the meta-learning phase, our model will work better, which will also lead to better practicality.

In our study, we only adopt simple Conv4 and Conv6 as feature extractors. In fact, SWE-MAML inherits the excellent nature of MAML. That is, it can be compatible with any CNN. One can adopt other more excellent CNNs, such as ResNet, and expect higher accuracy.

## 5 Conclusion

To realize few-shot crop disease recognition tasks in practical production, we propose a novel meta-learning model called SWE-MAML. We introduce an ensemble learning strategy to predict disease classes under the frame of MAML. We utilize MAML to train a set of base learners and their weights in sequence. When we train a base learner, the last already learnt base learner is used as its meta learner to provide the initialization parameter and do not need to train a separate meta learner for each base learner. Additionally, we propose a novel loss function that computes the loss of each base learner during the training process. The experimental results show that the method improves the current meta-learning model and demonstrates remarkable performance in few-shot crop disease recognition tasks. Compared to MAML, the recognition accuracy in the benchmark dataset PlantVillage improved by 3.75%-8.59%. The proposed method also shows higher average accuracy than the other FSL methods reported in recent years. Furthermore, our model is applied to the potato disease recognition task under natural conditions, with an accuracy of 75.71% when the size of the support set is only 30. The above results show that it is feasible to use SWE-MAML for few-shot crop disease recognition. The experimental results also indicate that Sequence-weighted ensemble dramatically reduces the variance of the recognition results.

By initializing each base-learner from its predecessor and then learning per-learner weights in the outer loop, SWE-MAML effectively constructs an adaptive ensemble inside the MAML framework. This both increases the diversity of task-specific hypotheses and retains the rapid-adaptation properties of gradient-based meta-learning. In effect, the weighted aggregation reduces variance in the meta-gradient estimate and smooths the loss landscape, yielding more stable convergence than a single-learner MAML. Unlike fixed ensemble schemes, our learned weighting dynamically emphasizes those base learners most suited to a new task. Theoretically, this can be viewed as performing a task-dependent model selection in the hypothesis space, which tightens the generalization bound by focusing capacity on the most relevant submodels.

In the natural-scene PDD benchmark, where images have complex backgrounds and lighting, SWE-MAML achieves 75.71% accuracy using only 30 support images per class—demonstrating its ability to generalize from clean lab data to “in-the-wild” scenarios. The method incurs only a linear increase in computation (proportional to the number of base learners) but does not require costly pre-training stages or external data. It can be plugged into any existing MAML codebase with minimal modifications, making it attractive for practitioners facing severe annotation constraints.

Overall, our method is outstanding for crop disease recognition tasks using very few annotated training images. This method provides a feasible solution for accurate disease recognition in scenarios where only a small number of disease samples are available. Meanwhile, the model’s performance needs to be improved in the cross-domain few-shot disease recognition task. For future studies, we plan to conduct more research on cross-domain few-shot disease recognition tasks.

## Data Availability

The original contributions presented in the study are included in the article/supplementary material. Further inquiries can be directed to the corresponding author.
